# The prevalence of mathematical difficulties among primary school children in Mainland China: a systematic review and meta-analysis

**DOI:** 10.3389/fpubh.2023.1250337

**Published:** 2024-02-08

**Authors:** Yanjiao Wang, Jia Long, Pei Wang

**Affiliations:** ^1^Faculty of Education, East China Normal University, Shanghai, China; ^2^College of Medical Humanities and Management, Wenzhou Medical University, Wenzhou, China; ^3^Key Research Center of Philosophy and Social Sciences of Zhejiang Province, Institute of Medical Humanities, Wenzhou Medical University, Wenzhou, China

**Keywords:** primary school children, mathematical difficulties, systematic review, meta-analysis, mathematics disabilities

## Abstract

The prevalence of mathematical difficulties (MD) among primary school children in China and its related factors remain unclear. To provide a suitable foundation for formulating appropriate screening and intervention strategies, this study aimed to estimate the prevalence of MD and its possible factors among primary school children in China. Articles were identified from seven databases and included if they reported on the prevalence of MD among primary school children in China. A total of 30,881 articles were retrieved, 975 of which were subjected to full evaluation. In total, 54 studies involving 34,815 participants (mean age 9.86 ± 3.13 years) were included. Analyses were conducted using the Meta and Metafor packages in the statistical software environment R. We used meta-regression and subgroup analyses by variable type to evaluate the potential prevalence factors. The pooled prevalence of MD among primary school children in China was 8.97%, whereas the prevalence rates of MD in lower, middle, and upper primary schools were 11.57%, 10.07%, and 4.76%, respectively. The results showed that the prevalence of MD among primary school children in China was significantly related to grade level and the cut-off points selected for screening MD and that the prevalence of MD in middle primary school was higher in girls than in boys. The prevalence of MD in primary schools in China was slightly higher than that in countries such as the United Kingdom and the United States. The prevalence of MD was higher in the lower and middle grades of primary school. The grade level and cut-off points selected during MD screening were closely related to the prevalence of MD. More attention should be paid to girls with MD in middle primary school.

**Systematic review registration:**

https://www.crd.york.ac.uk/prospero/display_record.php?ID=CRD42023410311, identifier: CRD42023410311.

## 1 Introduction

“Mathematical difficulties” (MD), also known as mathematics disabilities or dyscalculia (1), is a term used to describe individuals with an average or higher IQ and adequate educational resources but with extensive cognitive deficits in numerical cognition, attention, executive function, and other aspects (2). MD can affect children through the middle school level or even longer, and it can not only seriously influence the children's academic achievement, peer interaction, and social adaptation (3, 4) but also impact the country's economic development (5). Therefore, dealing with the challenge of MD has received considerable attention in various countries and fields worldwide.

However, the extent of the prevalence of MD in China remains unclear. Some researchers have reported that the prevalence of MD in China is consistent with that in the United States, the United Kingdom, and other countries, accounting for approximately 3.3%−6% of the total child population (6, 7); other studies, by contrast, have reported that this prevalence in China is as high as 13.8% (8). Therefore, in the absence of a consensus (9), further research is necessary to determine the prevalence of MD among Chinese children.

Chinese researchers have mainly relied on the Diagnostic and Statistical Manual (DSM-5) of the American Psychiatric Association (10) and the International Classification of Diseases (ICD-10) (11) when screening for MD (7, 8). Notably, the DSM-5 and ICD-10 classify MD as a specific learning disorder and developmental learning disorder, respectively and have several common criteria for defining MD, such as the significance/severity of the disorders. However, these two criteria differ in terms of defining MD. For example, the DSM-5 emphasizes that deficits in children with MD require parents to report that their child has various mathematics learning difficulties in school for at least 6 months (10). To effectively identify children with MD, Chinese researchers have followed the criteria of both the DSM-5 and ICD-10: excluding factors such as low intelligence and insufficient education and selecting standardized tools to test children who have significant differences in mathematical academic performance compared to typical children (7, 12). However, the tools used to measure MD vary widely. For example, some researchers have chosen standardized Chinese testing tools for screening. These tools primarily include the Chinese version of the Heidelberg Mathematics Ability Test (12), Chinese children and adolescents' mathematics academic achievement tests, screening tests for learning difficulties, and midterm and/or final examination scores in China (13). The Heidelberg Mathematics Ability Test is a scale developed by Chinese scholars to assess the basic mathematical abilities of Chinese children, after introducing and revising the “Heidelberg University Primary School Students' Basic Mathematical Abilities Test Scale” from Germany (14, 15). Currently, the Heidelberg Mathematics Ability Test has been widely used to test the mathematical abilities of Chinese children (12, 16). The midterm and final exams for children at the middle and end of each semester in China include mathematics and other tests, which have standardized criteria and are administered according to a systematic, scientific, and standardized procedure; therefore, they are considered to be standardized tests. Consequently, whether the choice of Chinese tools and the application of midterm and final examinations affect an accurate determination of the prevalence of MD remains unclear.

Using the DSM-5 and ICD-10 to determine the prevalence of MD in children, Chinese researchers have often chosen cut-off points to define MD, which is in line with practice in countries such as the United States and the United Kingdom (17). While the use of the cut-off point method distinguishes the severity of MD, it is also arbitrary (18). The cut-off point is based on a standardized assessment, and only children who score below a certain value are deemed to have MD. However, cut-off points have varied between studies. For example, some studies have defined the cut-off point as more than 1.0 standard deviation below the average score of normal children (19). Others have used cut-off points of more than 1.5, 2.0, and 3.0 standard deviations below the average score of normal children (8). This variation has resulted in different numbers of children being diagnosed with MD during screening. Therefore, in this study, the cut-off point selected during screening was used as a continuous variable to explore the effect of cut-off points on the prevalence of MD in China. Additionally, Chinese researchers have identified children with MD by obtaining information from their head or mathematics teachers, believing that this is a key aspect of using the DSM-5 in screening. Therefore, this study considered teacher participation in the screening of children with MD as an important variable to investigate its effects on the prevalence of MD in China.

Studies have found that the prevalence of MD in China may change with the age of children. In China, a large number of children with MD in the lower grades of primary schools have learning deficits, mainly due to their poor calculation skills; however, when they enter the middle or upper grades of primary schools, their calculation skills improve significantly (20). Nonetheless, some researchers have found that MD persists as children develop and becomes even more severe as they advance in school grades, such that the differences between them and typically developing children are amplified (21). Therefore, we conducted a meta-analysis to further investigate variations in the prevalence of MD by grade in China.

Additionally, studies have found significant sex differences in MD, with no consensus. For example, Li et al. (20) found that boys were more likely than girls to suffer from MD, while other studies have found the opposite; still others found no significant differences in the prevalence of MD between boys and girls (22). Thus, in this study, we evaluated whether the sex of children with MD affected its prevalence by performing a meta-analysis.

The comorbidity between MD and reading difficulties (RD) is also an important factor affecting prevalence. Children with combined MD and RD account for 80% of those with MD or RD (17). Some studies have shown that while children with RD or MD only have deficits in reading or mathematics, respectively, children with both MD and RD have more severe cognitive deficits, resulting in a higher prevalence of comorbidities in these children. However, Zhang et al. found that Chinese children with MD only had the same deficits as those who have both MD and RD and that Chinese children with MD and RD did not have more severe cognitive deficits than children with only MD (23). Therefore, this study used the presence of MD and RD as categorical variables to investigate whether they moderate the prevalence of MD in China.

In short, this study used a meta-analysis to investigate the prevalence of MD in China and the possible factors influencing its inconsistent prevalence, providing an important basis for the development of appropriate intervention strategies. Based on the findings, we discuss possible influencing factors and related theories in detail.

## 2 Methods

### 2.1 Literature retrieval

This meta-analysis has been registered (CRD42023410311), and the registration information can be found at https://www.crd.york.ac.uk/prospero/display_record.php?ID=CRD42023410311. This meta-analysis is reported according to the Preferred Reporting Items for Systematic Reviews and Meta-Analyses (PRISMA) guidelines. The study was conducted through a comprehensive search of all Chinese and English literature before November 2021. Chinese literature was searched in China National Knowledge Infrastructure (CNKI), the Wan Fang Database for Chinese Periodicals, and Weipu (CQVIP), and English literature was searched in APA PsycInfo, APA PsycArticles, Medline, and ERIC ((math^*^ AND difficult^*^ OR disability^*^) OR dyscalculia^*^) and (China^*^ OR Chinese^*^ OR Cantonese) were used as the keywords.

### 2.2 Inclusion and exclusion criteria

Two authors (WYJ and LJ) independently selected, retrieved, and assessed potentially relevant articles. The following criteria were used to select studies for inclusion in this study: studies that included children with MD in primary schools in China; studies that provided unambiguous data on the total sample sizes of typically developing children and children with autism, including information on how many autistic children were ultimately tested; in the MD screening phase of the studies, it was clear which tools were used to measure mathematics ability, whether teachers participated in the screening, and whether the IQ of children with MD was normal; studies where the cut-off point chosen for MD screening was clear; studies in which the control group of typical children was included; studies in which the final sample size of children with MD, including the numbers of boys and girls, was indicated; and research articles which were written in Chinese or English.

We excluded studies that did not report the number of samples used to screen children with MD, studies that did not have reports of cut-off points, studies that did not show whether the IQ of the children with MD was normal, and studies with no control group. Additionally, if the same author applied the same group of children with MD as participants to conduct multiple studies, only the most detailed study was retained to avoid double counting of the data.

### 2.3 Data extraction, coding, and inter-rater reliability

Two authors (WYJ and LJ) independently extracted data from the included studies and recorded the data using Excel. The extracted information included the following items: basic descriptive information (e.g., first author's name and publication year), variables involved in the screening approaches, and screening sample characteristics.

In particular, for extracting and coding grade information, we followed the Chinese grade levels: lower elementary school (grades 1–2, 7–8 years old), middle elementary school (grades 3–4, 9–10 years old), and upper elementary school (grades 5–6, 11–12 years old), for a total of three levels. For studies reporting that participants were recruited from discontinuous grades (e.g., first, third, and fifth grades), we first calculated the mean grade level and then coded it to the corresponding grade level, as described above, based on the mean grade level; for studies that did not report information on grade level, we estimated the grade level based on the mean age (e.g., a child with a mean age of 9.5 years would be considered to be attending middle elementary school in China).

In coding the remaining variables, we coded continuous variables, such as the cut-off point chosen for screening for MD, directly based on the cut-off point reported in each study; however, there were exceptions, such as coding the gender ratio, for which we calculated the gender ratio based on the number of male and female in the sample of the screened children with MD reported in the literature—for studies that did not report male and female, we uniformly coded the gender ratio as “no report.” Categorical variables, such as the presence of comorbidities with dyslexia in children with MD, were coded as “yes” (comorbidities with dyslexia) and “no” (no comorbidities with dyslexia).

Across the total variable matrix, the mean inter-rater agreement was 0.96, ranging from 0.92 to 0.98 for all variables investigated in this study. Disagreements were resolved through discussions.

### 2.4 Statistical analysis

R version 4.2.2 and the Meta and Metafor packages were used to analyze prevalence, map forests, and conduct Egger's test for publication bias. The prevalence of MD in children was used as the event rate, which mainly involved the sample size of the reported MD in each study and the total sample size at screening for MD. Due to the low prevalence in the included studies, the prevalence was pooled after a normal test using logit transformation. Sensitivity analyses, in which ρ varied between 0 and 1, were also conducted to further assess the estimated pooled prevalence, the standard error of its estimation (SE), and the between-study variance (Tau2). In addition, a heterogeneity test was applied if the p-value of the *Q* test was < 0.05 and I^2^ was more than 50% (24), which indicated that there was a high degree of heterogeneity among the studies; thus, a random effects model was used (25). The effect estimates are depicted in forest plots as proportions with 95% confidence intervals (95% CI).

In the specific analysis, we first estimated the overall prevalence and heterogeneity of MD in children in China. Considering that the prevalence of MD in different grades may differ, we divided the children into three subgroups according to grade level, namely, lower, middle, and upper grades of primary school, to further clarify the prevalence and influencing factors in different grades. Covariate analyses were performed when significant heterogeneity was observed among the four subgroups. Meta-regression analyses were used to test the relationship between prevalence and continuous variables, such as cut-off points. Moderating effect analyses were used to test the relationship between prevalence and categorical variables, such as the presence of comorbidities with RD (yes vs. no). When analyzing the moderating effect of categorical variables, the first category of each variable was used as a reference. For example, in the analysis of whether teacher participation affected prevalence, “yes” was used as the reference group.

## 3 Results

### 3.1 Description of the study

**Figure 1:**
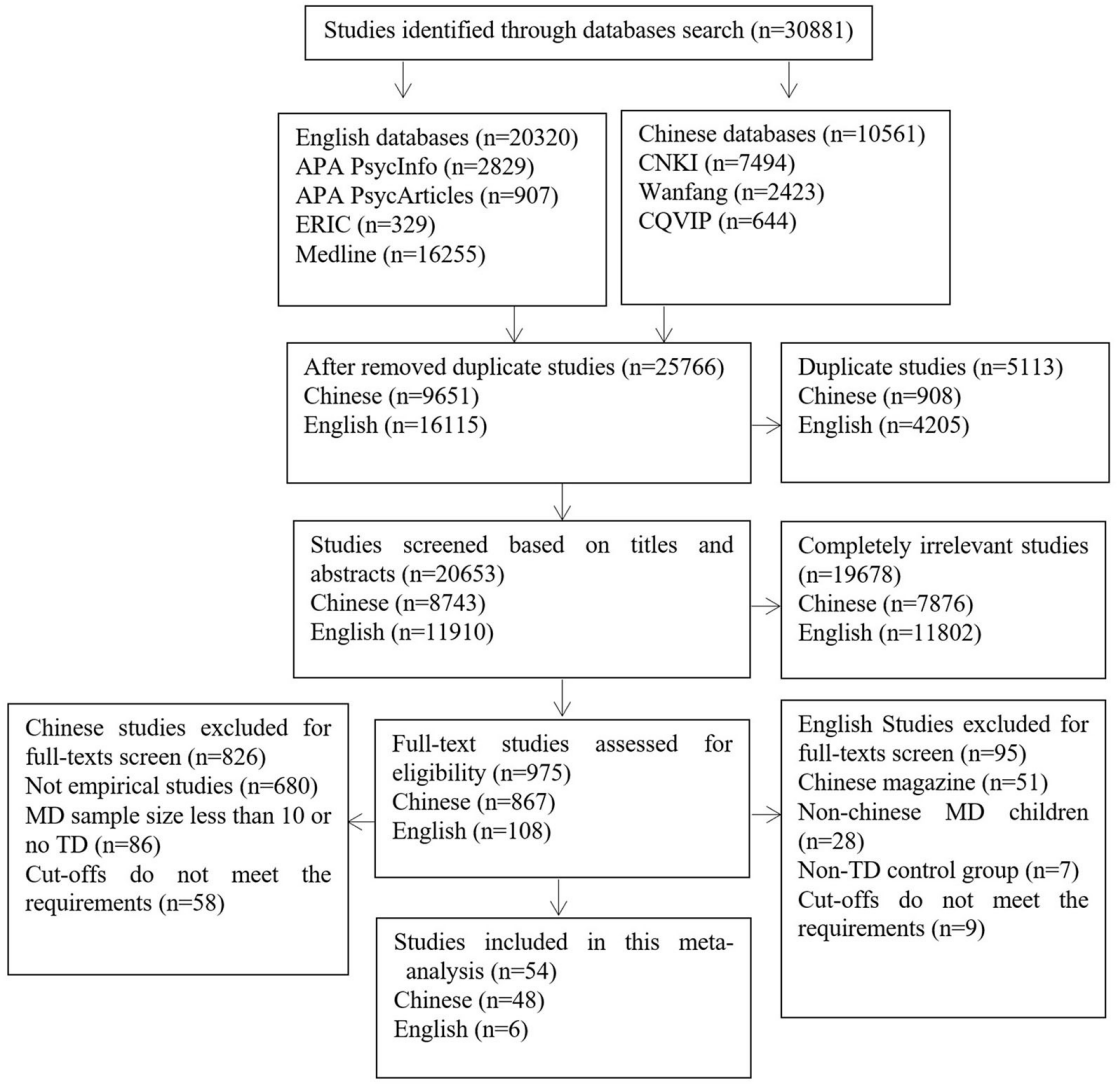
Literature screening process. Flowchart of the study selection process for the systematic review and meta-analysis following the guidelines from the Preferred Reporting Items for Systematic Reviews and Meta-Analyses (PRISMA) group.

A total of 10,561 articles were retrieved from Chinese databases. Among them, 7,494 articles were retrieved from CNKI, 2,423 articles were retrieved from the Wanfang Database, and 644 articles were retrieved from CQVIP. A total of 20,320 articles were retrieved from English databases. Among them, APA PsycInfo produced 2,829 English articles, APA PsycArticles produced 907, Medline produced 16,255, and ERIC produced 329. The specific screening process is shown in Figure 1. Two studies involved preschoolers; thus, they were not included. Table 1 shows the 54 studies and reflects those that were eventually included in the meta-analysis (7, 8, 12, 20, 22, 26–74). These studies include 24 journal papers (six in English) and 30 dissertations with a total of 34,815 participants (i.e., 2,727 children with MD screened from 34,815 among children).

**Table 1:** Baseline characteristics of included studies.

**References**	**Publish types**	**n_Total_**	**n_MD_**	**Grades**	**Screening approaches**			**Sample characteristics**	
					**Tools**	**Cut-off point**	**Teacher participation**	**Sex ratio**	**Comorbidity**
Cai et al. (26)	J	380	55	U	S	0.20	No	1.00	No
Ding et al. (27)	J	183	32	M	C	0.25	No	No report	No
Lin et al. (28)	J	237	82	M	C	0.25	No	No report	No
Huang and Chen (22)	J	805	38	M	C	0.25	No	1.05	Yes
Wang (29)	J	1,160	30	M	C	0.20	No	1.53	Yes
Zhang (30)	J	1,148	54	M	S	0.20	Yes	1.50	No
Cai (31)	D	260	20	L	S	0.20	Yes	1.45	No
Chen et al. (32)	J	300	32	L	S	0.25	Yes	2.33	No
Chen et al. (33)	J	600	60	U	S	0.20	Yes	1.00	No
Cheng and Gong (34)	J	1,564	66	M	S	0.12	Yes	No report	Yes
Du (35)	D	866	34	M	S	0.32	Yes	1.13	No
Han et al. (36)	J	450	84	L	S	0.25	No	1.71	No
He (37)	D	417	14	U	C	0.19	Yes	1.00	No
Jiao (38)	D	218	45	M	S	0.20	Yes	0.73	No
Lai et al. (39)	J	1,147	229	M	S	0.25	No	No report	No
Li (40)	D	1,177	97	U	S	0.10	Yes	No report	No
Li (41)	D	1,600	80	L	S	0.25	Yes	1.16	No
Li et al. (20)	J	2,057	100	M	C	0.00	No	1.44	No
Liu (42)	D	268	56	L	S	0.25	No	1.33	No
Liu (43)	D	1,083	36	M	S	0.12	Yes	1.40	No
Liu (44)	D	151	42	M	S	0.19	Yes	1.43	No
Liu and Cai (45)	J	160	39	M	S	0.20	No	0.93	No
Liu (46)	J	576	28	M	S	0.25	Yes	No report	No
Shang (47)	D	257	66	M	S	0.25	Yes	No report	No
Shi (48)	D	51	14	L	C	0.25	No	1.00	No
Tang (49)	D	417	14	U	S	0.12	Yes	1.00	No
Wan et al. (50)	J	350	57	M	S	0.25	Yes	0.90	Yes
Wang (51)	D	324	83	L	C	0.30	No	No report	No
Wang (52)	D	1,092	21	U	S	0.12	Yes	2.47	Yes
Wang (53)	D	210	33	L	S	0.25	Yes	1.20	No
Wang (54)	D	300	180	M	S	0.25	Yes	No report	Yes
Wang et al. (55)	J	87	45	M	S	0.30	Yes	No report	No
Wang (56)	D	810	15	M	S	0.25	Yes	2.57	No
Wu et al. (57)	J	703	48	M	S	0.10	No	0.92	No
Xiao (58)	D	325	24	M	S	0.20	Yes	No report	No
Xing et al. (59)	J	214	48	L	S	0.30	Yes	1.29	No
Xu (60)	D	530	24	L	S	0.15	No	1.00	No
Xu (61)	D	1,282	14	U	S	0.10	No	1.00	No
Xu (62)	D	408	30	M	S	0.12	Yes	No report	No
Xu (63)	D	128	20	M	S	0.20	Yes	No report	No
Xu (64)	D	408	38	L	S	0.12	Yes	No report	Yes
Yang et al. (7)	J	657	40	U	S	0.25	Yes	1.25	No
Ye (65)	D	1,224	74	L	S	0.25	Yes	0.68	No
Zhang (66)	D	294	49	M	S	0.10	Yes	1.42	No
Zhang et al. (8)	J	1,696	141	M	C	0.25	No	2.00	Yes
Zhang et al. (12)	J	411	19	L	C	0.25	No	0.73	No
Zhang and Zhang (67)	J	330	30	M	S	0.25	Yes	1.31	No
Zhang (68)	D	479	30	M	S	0.10	Yes	1.00	No
Zhang et al. (69)	J	2,364	46	M	S	0.10	Yes	1.56	No
Zhang (70)	D	381	20	U	S	0.10	Yes	1.00	No
Zhou (71)	J	407	17	U	S	0.19	No	0.23	No
Zhou (72)	D	827	42	U	S	0.10	Yes	0.83	No
Zhu and Wang (73)	J	700	16	M	S	0.25	Yes	1.82	Yes
Zuo (74)	D	342	76	M	S	0.20	Yes	1.62	No

J, journal articles; D, dissertation; L, lower grades of primary school; M, middle grades of primary school; U, upper grades of primary school; Tools, Math ability test tools; C, Chinese version of tools; S, middle/end of semester at school level or above; Teacher participation, whether teacher participation; Comorbidity, whether MD comorbidity with RD; RD, reading difficulties.

### 3.2 Pooled prevalence of MD in China

**Figure 2:**
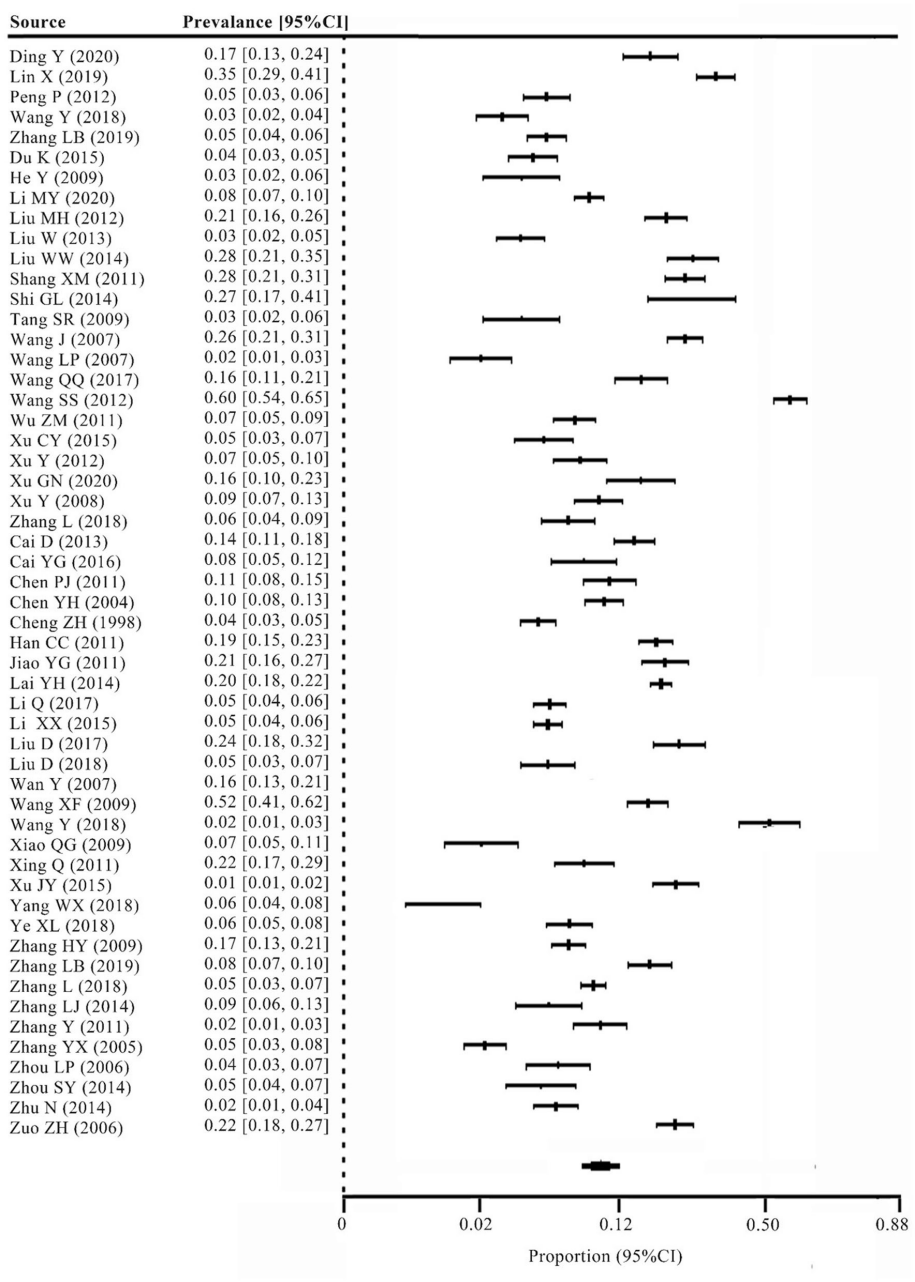
Forest plot of pooled mathematics difficulties prevalence among primary school children in China.

The pooled prevalence of MD in primary schools in China was 8.97%, 95%CI 0.07, 0.11. Figure 2 shows a forest plot of the pooled prevalence. Egger's test result was not significant (*t* = 1.99, *p* > 0.05), which indicated that there was no publication bias in this meta-analysis. Sensitivity analysis showed that the impact of the assumed within-study correlation between multiple effects (ρ) on the pooled prevalence, SE, and Tau^2^ was negligible for all outcomes. Heterogeneity test results showed that I^2^ = 97.61%, *Q* = 2,219.45, and *p* < 0.001. This indicated high heterogeneity; thus, it is necessary to use a random effects model to further analyze potential variables related to prevalence.

### 3.3 Influence of children's grades on the prevalence of MD in China

As mentioned above, to further explore the key population of MD in China, we divided the children with MD in China into three subgroups according to grades: lower grades of primary school, middle grades of primary school, and upper grades of primary school. The results (Table 2) showed that 13 studies were included in the lower grades, and the sample size of screening for MD was 6,250, yielding a pooled prevalence of MD of 11.57%, 95%CI [0.08, 0.17], *Q* = 285.15, *p* < 0.001, *I*^2^ = 95.79. In total, 30 studies were included in the middle grades; the sample size of screening for MD was 20,928, yielding a pooled prevalence of MD of 10.07%, 95%CI [0.07, 0.14], *Q* = 1,654.58, *p* < 0.001, *I*^2^ = 98.25. A total of 11 studies were included in the upper grades of primary school; the sample size of screening for MD was 7,637, yielding a pooled prevalence of MD of 4.76%, 95%CI [0.03, 0.07], *Q* = 155.21, *p* < 0.001, *I*^2^ = 93.56. The prevalence among the three subgroups of lower, middle, and upper grades was significantly different, *F*_(2, 51)_ = 3.29, *p* < 0.05. Therefore, we conducted further meta-regression analysis and moderating effect analysis for these three subgroups to explain the variability.

**Table 2:** Subgroup analysis based on grades.

**Grade**	***k***	**Prevalence**	**95%CI**	***Q***	***I* ^2^**	***F***
Lower grades	13	11.57%	0.08, 0.17	285.15^***^	95.79	3.29 (1, 51)
Middle grades	30	10.07%	0.07, 0.14	1,654.58^***^	98.25	
Upper grades	11	4.76%	0.03, 0.07	155.21^***^	93.56	

k is no. of studies, the same below. ^***^p < 0.001.

### 3.4 Impact of screening approaches on the prevalence of MD in China

All of the included literature (*k* = 54) reported the tools used to measure mathematical ability. In general, we found that tools were not significant factors affecting the prevalence of MD in China, *B* = −0.05, *p* > 0.05, *SE* = 0.37, 95%CI [−0.78, 0.69], *t* = −0.13. Further analysis showed that the prevalence of three subgroups did not change with the change of tools (all *p* > 0.05).

Based on the cut-off points selected for screening children with MD, we found that the cut-off point was an important factor affecting the prevalence of MD, *B* = 6.53, *p* < 0.01, *SE* = 1.89, 95%CI [2.73, 10.32], *t* = 3.45. The prevalence of MD in the lower, middle, and upper grades of primary school did not change with the change in the cut-off point, all *p* > 0.05.

From the perspective of prevalence and teacher participation in screening, in general, we found that whether teachers participated in screening did not affect the prevalence, *B* = 0.16, *p* > 0.05, *k* = 56, *SE* = 0.30, 95%CI [−0.45, 0.76], *t* = 0.52. The prevalence of MD among the three subgroups did not change with the change in teacher participation, all *p* > 0.05.

### 3.5 Impact of screening sample characteristics on the prevalence of MD in China

In terms of the relationship between prevalence and the sex ratio of children with MD, the sex ratio did not affect the prevalence rate, *B* = −0.25, *p* > 0.05, *k* = 41, *SE* = 0.31, 95%CI [−0.87, 0.37], *t* = −0.82. Further analysis showed that there was no significant gender difference in the prevalence of MD in lower or upper grades, both *p*-values were > 0.05. Only the middle grades of primary school had a significant gender difference in the prevalence of MD, *B* = −1.03, *p* < 0.05, *k* = 18, *SE* = 0.49, 95%CI [−2.06, −0.01], *t* = −2.12.

Regarding the prevalence and presence or absence of comorbidities with RD in children with MD, in general, we found that the presence or absence of comorbidities with RD did not affect prevalence, *B* = −0.33, *p* > 0.05, 95%CI [−1.09, 0.43], *t* = −0.88. The prevalence of each subgroup did not change with the change in comorbidities; all *p* > 0.05.

## 4 Discussion

This study examined the prevalence and possible factors influencing MD among primary school children in China. To the best of our knowledge, this is the first meta-analysis on the prevalence of MD in China. After the inclusion of 54 studies, it was found that the prevalence of MD in China was 8.97%, with 34,815 children screened, indicating that approximately 1 in 11 children had MD. The prevalence of MD decreased with an increase in the children's grades, and the critical turning point in the prevalence of MD occurred in the middle grades of primary school. The prevalence of MD in girls in the middle grades of primary school was higher than that in boys. The prevalence of MD was associated with the cut-off point chosen during screening.

### 4.1 Prevalence of MD in China and its influencing factors

Although previous studies have suggested that the prevalence of MD among elementary school children in China is 3%−6% (7, 20), we applied a meta-analysis and found a slightly higher prevalence than that reported in previous studies, ranging from 3% to 13.8% in some studies. This result was slightly higher than the prevalence of MD in countries such as the United States and the United Kingdom (8.97% and 6%, respectively) (75). This may be related to the relative shortage of special education resources and teachers in China. Compared to well-established special education support systems in countries such as the United Kingdom and the United States, China's special education system is undergoing improvement. This comparative lack of specific special capabilities combined with distinctive regional characteristics may be an important reason for the slightly higher prevalence of MD in China.

This study found that the prevalence of MD in the lower, middle, and upper grades of Chinese primary schools decreased as the children's grades increased. This result is consistent with the findings of Bai and Zang as well as previous research results from the 1990s (76, 77). This may be closely related to the fact that China emphasizes computational accuracy from an early age (78) and pays close attention to children's numerical performance and mathematical achievement. The finding that the prevalence of MD decreased as children's grade levels increased was consistent not only across cultures but also across time and space. Notably, we did not find any effect of the instruments and cut-off points used in screening on the prevalence rates at the lower-, middle-, or upper-grade levels. In general, cut-off points affect prevalence (79); however, cut-off points did not affect the prevalence of MD at each grade level. This suggests that the prevalence rate is related to that for the entire elementary school grade level. The main factors affecting the prevalence of certain grades remain to be further investigated.

Notably, there was a significant difference by sex in the prevalence of MD in the middle grades of primary school, with a higher prevalence in girls than in boys; this difference did not exist in other grades and is inconsistent with previous studies. For example, Li et al. (20) found that the prevalence of MD was higher in boys than in girls in the first and sixth grades of primary school. The most important reason may be that in the study by Li et al. (20), the participants were only recruited from a primary school in Beijing, China, and the sample size was small (100 children with MD in grades 1–6). In addition, previous studies have concluded that, because most students in the middle grades of elementary school are adolescents, gender differences in mathematical ability are related to biological reasons and that boys are more likely to choose mathematics-related courses and extracurricular activities (80). Moreover, previous studies have found that children who struggle with mathematics have higher mathematics anxiety than normal children and that there is a stronger negative correlation between mathematics anxiety and mathematics achievement in girls than in boys (81); therefore, boys might be expected to perform better in mathematics and be less likely to have MD than girls. Thus, the sex differences in the prevalence of MD in this age group in China found in this meta-analysis study are more likely to be representative.

The cut-off point selected for screening MD was found to be the main factor determining the prevalence of MD in China. This finding is similar to that of previous studies (82). That is, the prevalence of MD varies according to the cut-off values. For example, Swanson et al. used American children as participants and classified the cut-off point in terms of children with standardized mathematics test scores lower than 11% as having MD. However, that study did not include Chinese participants. According to a large number of studies (83), children with scores between 25% and 40% are at risk of developing MD. Therefore, this study included all children with standardized mathematics ability test score cut-off points to comprehensively analyze the relationship between the prevalence of MD and the cut-off point in China and found that the cut-off point affected the prevalence of MD. This suggests that the relationship between the prevalence of MD in children and the cut-off point chosen for screening may be consistent across cultures.

This study had several limitations. First, the literature selected for the meta-analysis in this study was cross-sectional, which precluded accurate determination of related influencing factors. Second, the relationship depicted using meta-regression is an observational association across trials and is not suitable for explaining causality (84). Finally, although we attempted to assess the influence of sample characteristics on the prevalence of MD in China, certain possible factors, such as IQ, were not considered (85). Previous research has found significant differences in intelligence between children with MD and normal children (85); additionally, in the field of dyslexia, one of the most influential factors in its prevalence is the criterion of intelligence (86), suggesting that intelligence may affect the likelihood of children having MD in terms of screening results (86) and the incidence of children with MD (17). Therefore, future research should incorporate more variables, such as IQ, into screening for MD (e.g., using standardized score comparisons) (87), which, in turn, could influence the incidence of MD in China.

### 4.2 Implications and future directions

This study is the first to identify the prevalence of MD among primary school children in China. Its findings can help guide research on MD in China in terms of focusing on the reasons behind its prevalence and identifying appropriately targeted interventions for children with MD to reduce its prevalence.

The prevalence of MD in China was highest in the lower grades of primary school and decreased as the grades increased. This may be related to China's emphasis on calculation skills beginning in early childhood (78) and its focus on children's numerical performance and mathematical achievement. However, this general approach overlooks differences among children and the relationship between other cognitive skills and mathematical abilities. It is well known that there are different subtypes of MD (88). Moreover, a large number of studies have shown that working memory, visual-spatial skills, and learning strategies play an important role in children's mathematics learning (2, 89) and that these abilities (especially phonological processing and working memory) have a major influence on children with MD (2). Thus, this study recommends that future studies provide specific guidance to help teachers and parents implement personalized education and training based on clarifying subtypes or individual differences in relation to MD in China, as well as help identify specific deficits in children related to working memory and executive function to improve children's mathematical ability and reduce the prevalence of MD.

Furthermore, Chinese researchers in the field of MD must be encouraged to develop an early recognition and intervention system based on artificial intelligence; establish an early recognition system for MD through machine learning and other technologies; and vigorously promote an adaptive training and personalized guidance system to provide extensive and intensive support in relation to MD based on artificial intelligence technology. This would enable teachers, schools, and families, especially those who lack funds, to receive personalized special education resources with appropriate feedback and intervention. Early identification and intervention methods based on artificial intelligence are receiving increasing attention. Although little attention has been paid to interventions in relation to MD, many researchers in the field of RD research believe that machine learning can not only quickly and objectively identify RD but also improve the accuracy of screening and distinguish the subtypes of RD by integrating multi-modal indicators of children's behavior and neurological activity characteristics (90) to improve intervention quality (91, 92). We found that parents and teachers of children with MD in China have limited abilities to identify children with MD and that they lack special education resources, resulting in a lack of intervention for these children. Therefore, the development of an early identification and intervention system based on artificial intelligence is likely to not only be conducive to the early diagnosis of MD but also enable evidence-based training to improve intervention effectiveness and reduce the prevalence of MD. This may be a relatively effective method, and future studies should attempt to verify it.

The results of this study can serve as a reference for relevant authorities when formulating MD screening and interventions. For example, a country might provide support to its children in the following ways. First, increase awareness of MD and use appropriate identification methods for identifying MD among communities, parents, and schools; this would facilitate early diagnoses and interventions for children with MD, especially in the lower and middle grades of primary school, and help reduce its prevalence. Second, increase the number of specialized diagnostic clinics for learning disabilities, including MD, in major hospitals to facilitate timely assessment by parents and teachers and carry out clinical intervention under the advice of professional doctors (93).

## 5 Conclusion

This meta-analysis is the first to estimate the prevalence of MD in China. The results suggest that the prevalence of MD in China is 8.97%. The prevalence was closely related to the cut-off points selected during MD screening. The middle grades of primary school were identified as the key turning point for the development of mathematical ability; during this stage, the prevalence of MD was shown to be influenced by sex, with a higher prevalence in girls than in boys.

## Data availability statement

The original contributions presented in the study are included in the article/supplementary material, further inquiries can be directed to the corresponding author/s.

## Author contributions

YW conceptualized and designed this study, designed the data collection instruments, collected data, carried out the initial analysis, drafted the initial manuscript, and reviewed and revised the manuscript. YW and JL collected data, carried out the initial analyses, and reviewed and revised the manuscript. PW conceptualized and designed the study, coordinated and supervised data collection, and critically reviewed the manuscript for important intellectual content. All authors approved the final manuscript as submitted and agree to be accountable for all aspects of the work.

## References

[1] Nelson G Powell SR. A systematic review of longitudinal studies of mathematics difficulty. J Learn Disabil. (2018) 51:523–39. 10.1177/0022219417714773 28613104

[2] Peng P Wang CC Namkung J. Understanding the cognition related to mathematics difficulties: a meta-analysis on the cognitive deficit profiles and the bottleneck theory. Rev Educ Res. (2018) 88:434–76. 10.3102/0034654317753350

[3] Morgan PL Farkas G Wang YY Hillemeier MM Oh Y Maczuga S. Executive function deficits in kindergarten predict repeated academic difficulties across elementary school. Early Child Res Q. (2018) 46:20–32. 10.1016/j.ecresq.2018.06.009

[4] Powell SR Doabler CT Akinola OA Therrien WJ Maddox SA Hess KE . A synthesis of elementary mathematics interventions: comparisons of students with mathematics difficulty with and without comorbid reading difficulty. J Learn Disabil. (2020) 53:244–76. 10.1177/0022219419881646 31631747

[5] Butterworth B Varma S Laurillard D. Dyscalculia: from brain to education. Science. (2011) 332:1049–53. 10.1126/science.1201536 21617068

[6] Szucs D Devine A Soltesz F Nobes A Gabriel F. Developmental dyscalculia is related to visuo-spatial memory and inhibition impairment. Cortex. (2013) 49:2674–88. 10.1016/j.cortex.2013.06.007 23890692 PMC3878850

[7] Zhang LB Zhang L Feng TY. Number sense deficits in children with developmental dyscalculia. Chin J Psychol Behav Res. (2019) 7:512–9.

[8] Yang WX Zhang TZ Li HX Zhang JJ Si JW. Central executive load effect of estimation strategy used in children with mathematical difficulties. Chin J psychol. (2018) 50:504–16. 10.3724/SP.J.1041.2018.00504

[9] Li DF Zhang XJ Zhang L. What skills could distinguish developmental dyscalculia and typically developing children: evidence from a 2-year longitudinal screening. J Learn Disabil. (2022) 8:222194221099674. 10.1177/00222194221099674 35674456

[10] American Psychiatric Association. Diagnostic and Statistical Manual of Mental Disorders: DSM-5 (5th ed.). Washington, D.C.: American Psychiatric Association (2013). 10.1176/appi.books.9780890425596

[11] World Health Organization. International Statistical Classification of Diseases and Related Health Problems (11th Revision). Geneva: World Health Organization (2018). Available online at: https://icd.who.int/browse11/l-m/en (accessed December 18, 2019).

[12] Zhang L Jiang H Zhao L. Quantitative conversion deficits in children with developmental dyscalculia. Chin J Psycholo Sci. (2018) 41:1671–6981. 10.16719/j.cnki.1671-6981.20180213

[13] Mammarella IC Toffalini E Caviola S Colling L Szucs D. No evidence for a core deficit in developmental dyscalculia or mathematical learning disabilities. J Child Psychol Psyc. (2021) 62:704–14. 10.1111/jcpp.13397 33684972

[14] Wu HR Li L John H. Research on the application of mathematics ability test for primary school students. Chin Sch Health. (2003) 4:331–333. 10.3969/j.issn.1000-9817.2003.04.035

[15] Wu HR Li L. Preparation and reliability and validity test of mathematical ability test scale for primary school students. Chin Public Health. (2005) 21:93–5. 10.11847/zgggws2005-21-04-58

[16] Kang D Wen M Zhang YJ. The relationship between fine motor skills and mathematical ability in children: a meta-analysis. Chin Adv Psycho Sci. (2023) 31:1443–59.

[17] Swanson HL Olide AF Kong JE. Latent class analysis of children with math difficulties and/or math learning disabilities: are there cognitive differences? J Educ Psychol. (2018) 110:931–51. 10.1037/edu0000252

[18] Kroesbergen EH Huijsmans MDE Friso-van den Bos I. A meta-analysis on the differences in mathematical and cognitive skills between individuals with and without mathematical learning disabilities. Rev Educ Res. (2022) 93:718–55. 10.3102/00346543221132773

[19] Kang D Li FY Wen X Hu Z Yang ZH Li J . Effects of 4-week play training on working memory in children with underlying mathematical learning difficulties aged 5-6 years. Chin J Ment Health. (2018) 32:495–501.

[20] Li XX Yang JX Lu H Wang F Zhao H. Basic quantitative processing deficits and general cognitive characteristics in developmental dyscalculia. Chin J Spec Educ. (2015) 8:56–63. 10.3969/j.issn.1007-3728.2015.08.009

[21] Basang ZM Tan RN Shi NZ. A comparative study of number sense between numerically poor and numerically superior students in Tibetan primary schools. J Math Educ. (2023) 32:30–6.

[22] Huang DQ Chen YH. Development of mathematical cognitive ability of children with mathematical difficulties in Grade 2 to Grade 6. Chin J Math Educ. (2016) 25:70–4.

[23] Zhang Y Tian MY Yang XM. The effect of picture information on problem solving: evidence from eye movements. China Spec Educ. (2022) 9:77–87.

[24] Higgins JPT Thompson SG Deeks JJ Altman DG. Measuring inconsistency in meta-analyses. Brit Med J. (2003) 327:557–60. 10.1136/bmj.327.7414.557 12958120 PMC192859

[25] Borenstein M Hedges LV Higgins JT Rothstein HR A. basic introduction to fixed-effect and random-effects models for meta-analysis. Res Synth Methods. (2010) 1:97–111. 10.1002/jrsm.12 26061376

[26] Cai D Li QW Deng CP. Characteristics of Working memory in Junior high School students with Math failure: domain generality or specificity? Chin J Psychol. (2013) 45:193–205. 10.3724/SP.J.1041.2013.00193

[27] Ding Y Liu RD Hong W Yu Q Wang J Liu Y . Specific mental arithmetic difficulties and general arithmetic learning difficulties: the role of phonological working memory. Psychol Rep. (2020) 124:720–751. 10.1177/0033294120916865 32295484

[28] Lin X Peng P Luo HJ. The deficit profile of elementary students with computational difficulties versus word problem-solving difficulties. Learn Disab Q. (2019) 44:110–22. 10.1177/0731948719865499

[29] Wang Y. The effect of math anxiety and working memory on word problem solving of children with math learning difficulties. Cent China Normal University (2018).

[30] Zhang SH. Effect of approximate number system training on primary school children with math learning difficulties. Jiangxi Normal University (2018).

[31] Cai YG. Study on SNARC Effect in children with mathematical difficulties under Different Quantitative information and Cognitive Control conditions. East China Normal University (2016).

[32] Chen PJ Zhang J Chen YH. A new perspective to explain the characteristics of working memory in children with mathematical difficulties – from the perspective of co-centered model. Chin J Spec Educ. (2011) 1:18–24. 10.3969/j.issn.1007-3728.2011.01.004

[33] Chen YH Zhao YQ Zhang KJ Wang MY. Comparison of addition strategies between 7-8 years old children with math learning difficulties and normal children. Chin J Spec Educ. (2004) 11:3–7.

[34] Cheng ZH Gong YX. A comparative study of memory in Children with Learning disabilities II Long-term memory function in children with learning disabilities. Chin J Clin Psychol. (1998) 4:26–31. 10.1088/0256-307X/16/12/025

[35] Du K. Research on the neuropsychological characteristics of information processing in children with developmental dyscalculia. Guangdong Pharm University (2015).

[36] Han CC Zhang J Huang DQ Chen YH. Comparison of quantitative estimation ability of children with mathematical difficulty in grades 2-4. Chin J Spec Educ. (2010) 118:47–51. 10.3969/j.issn.1007-3728.2010.04.010

[37] He Y. An experimental study on the characteristics of implicit self-esteem in children with learning disabilities. Hunan Normal University (2009).

[38] Jiao YG. A Study on the Cognitive Ability of primary school children with math learning difficulties based on the CHC theory. Shaanxi Normal University (2011).

[39] Lai YH Zhu XS Huang DQ Chen YH. Comparison of spatial ability of children with math learning difficulties and normal children in grades 3-6. Chin J Psychol Behav Res. (2014) 12:36–44.

[40] Li MY. Spatial Visualization ability of children with different subtypes of math learning difficulties in grades 4-6. Nanjing Normal University (2020).

[41] Li Q. Research on the causes and subtypes of developmental dyscalculia. Southwest University (2017).

[42] Liu MH. Early screening and dynamic intervention of children with poor number sense. Ningxia University (2012).

[43] Liu W. The influence of material types on the number cognition of children with dyscalculia. Hunan Normal University (2013).

[44] Liu WW. A study on the influence of endogenous and exogenous attention on the digital distance effect in children with mathematical difficulties. Huazhong University Science Technology (2014).

[45] Liu D Cai D. The development of number line estimation ability and its relationship with the cognitive processes of PASS in mathematically challenged students. Chin J Spec Educ. (2017) 12:32–8.

[46] Liu D. Research on the representation process of word problems in children with different subtypes of math learning difficulties. Chin Psychol Behav Res. (2018) 16:497–504.

[47] Shang XM. Study on the relationship between primary school students' mathematical learning difficulties and their inhibitory ability development. Liaoning Normal University (2011).

[48] Shi GL. Neural mechanism of basic numerical processing in children with different developmental levels of number sense. Ningxia University (2014).

[49] Tang SR. Effects of material properties on mental rotation of children with learning disabilities. Hunan Normal University (2009).

[50] Wan Y Tao DQ Liao SL. A study on working memory span of primary school children with math learning difficulties. Chin J Spec Educ. (2007) 7:46–51. 10.3969/j.issn.1007-3728.2007.07.010

[51] Wang J. Research on the separation of Elementary Mathematics Ability and Central executive control function of primary school students. Shaanxi Normal University (2007).

[52] Wang LP. Research on executive function of students with learning difficulties. Hebei University (2007).

[53] Wang QQ. Research on the influence factors and intervention of math core experience of children with poor math performance in primary school. Shaanxi Normal University (2017).

[54] Wang SS. A study on the reading comprehension characteristics and eye movement of 4th ~ *6th grade students with difficulty in mathematics learning*. Henan University (2012).

[55] Wang XF Liu XN Luo XY Zhou RL. A developmental study on inhibitory ability of children with mathematical disabilities. Chin J Spec Educ. (2009) 10:55–59. 10.3969/j.issn.1007-3728.2009.10.011

[56] Wang Y. The effect of math anxiety and working memory on word problem solving of children with math learning difficulties. Central China Normal University (2018).

[57] Wu ZM Huang Y Wang QX Zhou F Ma X Lin Mei J . The relationship between visuospatial working memory and mathematical ability in children with math learning disabilities. Chin J Child Health. (2011) 19:890–6.

[58] Xiao QG. A study on numerical cognitive deficits in children with MD. Southwest University (2009).

[59] Xing Q Cai XH Chen XX. Research on the characteristics of word problem representation of primary school students with difficulty in mathematics learning. Chin Educ Guide. (2011) 12:31–4. 10.3969/j.issn.1005-3476.2011.12.009

[60] Xu CY. ERPs study of different forms of quantitative representation and quantitative manipulation in children with poor number sense. Ningxia University (2015).

[61] Xu JY. An ERP study on visual spatial attention range and attention Transfer in children with learning Difficulties. Xinxiang Medical College (2015).

[62] Xu Y. Research on Addition and Subtraction of Children with Developmental dyscalculia. Hunan Normal University (2012).

[63] Xu GN. Zhao yang district a primary school learning difficulties student attention intervention research. Yunnan Normal University (2020).

[64] Xu Y. Characteristics of Word Problem Representation of Children with poor Mathematics Learning in Primary school. Henan University (2008)

[65] Ye XL. Mechanisms of Working memory and Quantitative Representation on arithmetic learning difficulties in primary school children. East China Normal University (2018).

[66] Zhang HY. Research on Cognitive Mechanism of Developmental dyscalculia in children. Huazhong University Science Technology (2009).

[67] Zhang LJ Zhang ZF. Further identification of children with “mathematical learning difficulties” by dynamic testing. Chin J Psychol. (2014) 46:1112–1123. 10.3724/SP.J.1041.2014.01112

[68] Zhang SH. Effect of Approximate number System Training on Primary school Children with Math learning difficulties. Jiangxi Normal University (2018).

[69] Zhang Y Liu AS Zhang XZ Zhang L. Verbal and visuospatial working memory in children with different subtypes of learning disabilities. Chin J Clin Psychol. (2011) 19:641–4. 10.16128/j.cnki.1005-3611.2011.05.036

[70] Zhang YX. Research on the estimation characteristics of children with math academic disabilities. Shandong Normal University (2005).

[71] Zhou SY. Symbolic short-term Memory extraction and mental rotation of children with learning disabilities. Hunan Normal University (2014).

[72] Zhou SY. Symbolic short-term Memory extraction and mental rotation of children with learning disabilities. Hunan Normal University (2014).

[73] Zhu N Wang Y. A study on the pattern characteristics and effectiveness of word problem solving process for children with math learning difficulties in the fourth grade of primary school. Chin J Spec Educ. (2014) 167:39–48. 10.3969/j.issn.1007-3728.2014.05.007

[74] Zuo ZH. Cognitive processing mechanism of primary school students' mathematics learning difficulties. East China Normal University (2006).

[75] Wilson AJ Andrewes SG Struthers H Rowe VM Bogdanovic R Waldie KE. Dyscalculia and dyslexia in adults: Cognitive bases of comorbidity. Learn Individ Differ. (2015) 37:118–32. 10.1016/j.lindif.2014.11.017

[76] Bai XJ Zang CL. Research on developmental dyscalculia and strategies for mathematics education. J Liaoning Norm Univ. (2006) 1:45–9. 10.3969/j.issn.1000-1751.2006.01.013

[77] Silver CH Pennett HL Black JL Fair GW Balise RR. Stability of arithmetic disability subtypes. J Learn Disabil. (1999) 32:108–19. 10.1177/002221949903200202 15499712

[78] Geary DC Bowthomas CC Fan L Siegler RS. Even before formal instruction, chinese children outperform american children in mental addition. Cogn Dev. (1993) 8:517–29. 10.1016/S0885-2014(05)80007-3

[79] Yang LP Li CB Li XM Zhai MM Zhao J Weng XC . Prevalence of developmental dyslexia in primary school children: a protocol for systematic review and meta-analysis. World J Pediatr. (2022) 18:804–9. 10.1007/s12519-022-00572-y 35759111

[80] Chen YH Zhong NN Tian GS Wang ZG. A study on the difference of representation strategies of mathematics word problems in grade 2~4 children. Chin Psychol Dev Educ. (2004) 4:19–24. 10.3969/j.issn.1001-4918.2004.04.004

[81] Barroso C Ganley CM McGraw AL Geer EA Hart SA Daucourt MC . Meta-analysis of the relation between math anxiety and math achievement. Psychol Bull. (2021) 147:134–68. 10.1037/bul0000307 33119346 PMC8300863

[82] Murphy MM Mazzocco MM Hanich LB Early MC. Cognitive characteristics of children with mathematics learning disability (MLD) vary as a function of the cutoff criterion used to define MLD. J Learn Disabil. (2007) 40:458–78. 10.1177/00222194070400050901 17915500

[83] Swanson HL. Word problem solving, working memory and serious math difficulties: do cognitive strategies really make a difference? J Appl Res Mem Cogn. (2016) 5:368–83. 10.1016/j.jarmac.2016.04.012

[84] Maraz A Griffiths MD Demetrovics Z. The prevalence of compulsive buying: a meta-analysis. Addiction. (2016) 111:408–19. 10.1111/add.13223 26517309

[85] Chu FW Vanmarle K Geary DC. Quantitative deficits of preschool children at risk for mathematical learning disability. Front Psychol. (2013) 4:195. 10.3389/fpsyg.2013.00195 23720643 PMC3655274

[86] Di Folco C Guez A Peyre H Ramus F. Epidemiology of reading disability: a comparison of DSM-5 and ICD-11 criteria. Sci Stud Read. (2022) 26:337–355. 10.1080/10888438.2021.1998067

[87] Clarizio HF Phillips SE. Defining severe discrepancy in the diagnosis of learning disabilities: a comparison of methods. J School Psychol. (1989) 27:383–97. 10.1016/0022-4405(89)90015-0

[88] Chan WWL Wong TTY. Subtypes of mathematical difficulties and their stability. J Educ Psychol. (2020) 112:649–666. 10.1037/edu0000383

[89] Yang X Chung KKH McBride C. Longitudinal contributions of executive functioning and visual-spatial skills to mathematics learning in young Chinese children. Educ Psychol. (2019) 39:678–704. 10.1080/01443410.2018.1546831

[90] Latifoglu F Ileri R Demirci E. Assessment of dyslexic children with EOG signals: determining retrieving words/re-reading and skipping lines using convolutional neural networks. Chaos Soliton Fract. (2021) 145:110721. 10.1016/j.chaos.2021.110721

[91] Atkar G. Speech synthesis using generative adversarial network for improving readability of Hindi words to recuperate from dyslexia. Neural Comput Appl. (2021) 33:9353–62. 10.1007/s00521-021-05695-3 33612979 PMC7883547

[92] Oliaee A Mohebbi M Shirani S Rostami R. Extraction of discriminative features from EEG signals of dyslexic children; before and after the treatment. Cogn Neurodyn. (2022) 16:1249–59. 10.1007/s11571-022-09794-2 36408072 PMC9666605

[93] Lin GX Chen S Chen DG Hu J Yin XR. Evaluation of the efficacy of comprehensive intervention in children with learning difficulties. Chin Clin Rehabil. (2005) 24:210–2. 10.1111/j.1365-4632.2004.02244.x

